# The *Campylobacter jejuni* RacRS two-component system activates the glutamate synthesis by directly upregulating γ-glutamyltranspeptidase (GGT)

**DOI:** 10.3389/fmicb.2015.00567

**Published:** 2015-06-05

**Authors:** Anne-Xander van der Stel, Andries van Mourik, Paweł Łaniewski, Jos P. M. van Putten, Elżbieta K. Jagusztyn-Krynicka, Marc M. S. M Wösten

**Affiliations:** ^1^Department of Infectious Diseases and Immunology, Utrecht University, UtrechtNetherlands; ^2^Department of Bacterial Genetics, Institute of Microbiology, Faculty of Biology, University of Warsaw, WarsawPoland

**Keywords:** *Campylobacter jejuni*, gene regulation, two-component system, RacRS, γ-glutamyltranspeptidase, GGT, glutamine-2-oxoglutarate aminotransferase, GOGAT

## Abstract

The highly conserved enzyme γ-glutamyltranspeptidase (GGT) plays an important role in metabolism of glutathione and glutamine. Yet, the regulation of *ggt* transcription in prokaryotes is poorly understood. In the human pathogen *Campylobacter jejuni*, GGT is important as it contributes to persistent colonization of the gut. Here we show that the GGT activity in *C. jejuni* is dependent on a functional RacRS (reduced ability to colonize) two-component system. Electrophoretic mobility shift and luciferase reporter assays indicate that the response regulator RacR binds to a promoter region ~80 bp upstream of the *ggt* transcriptional start site, which contains a recently identified RacR DNA binding consensus sequence. RacR needs to be phosphorylated to activate the transcription of the *ggt* gene, which is the case under low oxygen conditions in presence of alternative electron acceptors. A functional GGT and RacR are needed to allow *C. jejuni* to grow optimally on glutamine as sole carbon source under RacR inducing conditions. However, when additional carbon sources are present *C. jejuni* is capable of utilizing glutamine independently of GGT. RacR is the first prokaryotic transcription factor known to directly up-regulate both the cytoplasmic [glutamine-2-oxoglutarate aminotransferase (GOGAT)] as well as the periplasmic (GGT) production of glutamate.

## Introduction

The enzyme γ-glutamyltranspeptidase (GGT, EC 2.3.2.2) is highly conserved among eukaryotic and prokaryotic organisms (Ong et al., 2008), where it has a key function in glutathione metabolism. In prokaryotes GGT is produced as a proenzyme in the cytoplasm and is then translocated into the periplasm where it undergoes autocatalytic cleavage. This proteolysis yields a mature dimer which transfers γ-glutamyl moieties from extracellular glutathione and related compounds to amino acids or peptides or catalyzes the hydrolysis of the glutamyl group to generate glutamate (Hanigan, 1998). GGT activity in *Escherichia coli* and *Bacillus subtilis* is maximal in stationary growth phase (Suzuki et al., 1986; Xu and Strauch, 1996). In *B. subtilis* GGT is indirectly transcriptionally regulated in response to low L-glutamate concentrations via the quorum sensing two-component system ComP/ComA (Kimura et al., 2004). In *Helicobacter pylori* expression of GGT is reported not to be growth phase dependent, but is up-regulated at low pH (Wachino et al., 2010) and to be involved in acid resistance and immune stimulation (Gong et al., 2010; Miller and Maier, 2014).

The bacterium *C. jejuni* is a major food borne pathogen in humans and colonizes the intestinal tract of many warm-blooded animals (Blaser, 1997). *C. jejuni* lacks the glycolytic enzyme phosphofructokinase and is therefore not able to use exogenous sugars as a carbon source, although some strains were shown to be able to metabolize fucose (Muraoka and Zhang, 2011; Stahl et al., 2011). Hence, amino acids (i.e., aspartate, glutamate, proline, and preferentially serine) are likely to sustain the growth of *Campylobacter* in the intestine (Guccione et al., 2008). Although the genome of *C. jejuni* encodes for a functional glutamine transporter (Lin et al., 2009), only isolates containing GGT are also able to utilize glutamine, and glutathione as sole carbon/energy source (Hofreuter et al., 2006). In the periplasm this enzyme converts glutamine and glutathione to glutamate, which is subsequently taken up via the aspartate/glutamate-binding protein PEB1 (Del Rocio Leon-Kempis et al., 2006). After glutathione cleavage by GGT the remaining dipeptide cys-gly is also imported by *C. jejuni* and used as sulfur source (Vorwerk et al., 2014). The expression of *ggt* is reported to be maximal in late log phase (Hyytiäinen et al., 2012). The presence of GGT allows *C. jejuni* strains to enhance their colonization persistence in the avian gut and to colonize the intestine of mice (Barnes et al., 2007; Hofreuter et al., 2008).

The regulator RacR (reduced ability to colonize) is like GGT needed to sustain the colonization of chickens (Brás et al., 1999) and is detected in nearly all *C. jejuni* isolates (Kordinas et al., 2005; Talukder et al., 2008; Quetz et al., 2012). Recently we showed that the RacRS two-component system of *C. jejuni* is active under low oxygen conditions in the presence of alternative electron acceptors (e.g., nitrate or TMAO; van der Stel et al., 2015). Under these conditions, RacR represses the transcription of several genes including the *aspA* gene and at the same time it activates the *gltBD* genes. The products of the *gltBD* genes form the glutamine-2-oxoglutarate aminotransferase (GOGAT) complex, which is responsible for cytosolic glutamate generation (Guccione et al., 2008). Glutamate is an important nitrogen source for bacteria as it functions as precursor for amino acid and nucleotide anabolism (Reitzer, 2003; Heeswijk et al., 2013; Hofreuter, 2014). Here we investigated whether the generation of periplasmic glutamate accomplished by GGT is also regulated by RacR.

## Materials and Methods

### Bacterial Strains, Media, and Growth Conditions

Bacterial strains and plasmids used in this study are listed in **Table 1**. *C. jejuni* strains were cultured at 37°C or 42°C on Blood Agar Base No. 2 (BA) medium containing 5% horse blood or in Heart Infusion broth (HI; Oxoid), under microaerobic conditions (5% O_2_, 7.5% CO_2_, 7.5% H_2_, 80% N_2_), or under oxygen limited conditions (0.3% O_2_, 10% CO_2_, 10% H_2_, 80% N_2_). Kanamycin (25 μg ml^-1^) and/or chloramphenicol (15 μg ml^-1^) were added when appropriate. *E. coli* strains were routinely grown at 37°C in Luria-Bertani (LB) broth or on LB agar plates supplemented with ampicillin (100 μg ml^-1^), kanamycin (30 μg ml^-1^), or chloramphenicol (30 μg ml^-1^).

**Table 1:** Bacterial strains and plasmids used in this study.

Bacterial strains and plasmids	Relevant characteristics	Origin or reference
**Strains**
*Escherichia coli* TG1	*supE hsd*Δ*5 thi*Δ(*lac-proAB*) *F’* (*traD36 proAB*^+^ *lacI*^q^ *lacZ*ΔM15); used for general cloning	Green and Sambrook (2012)
*E. coli* BL21(DE3)	F^-^ *ompT hsdSB* (r_B_^-^ m_B_^-^) *gal dcm* (DE3); used for protein overexpression	Novagen
*E. coli* S17	*recA pro hsdR* RP4-2-Tc::Mu-Km::Tn*7* (Tmp^r^ Str^r^); used for conjugation	Parke (1990)
*Campylobacter jejuni* 81116	Wildtype	Palmer et al. (1983)
*C. jejuni ggt*	81116 derivative *ggt*::Cm	This study
*C. jejuni racR*	81116 derivative *racR*::Cm	van der Stel et al. (2015)
*C. jejuni racS*	81116 derivative *racS*::Cm	This study
*C. jejuni racRS*	81116 derivative *racRS*::Cm	This study
*C. jejuni racR+*p	81116 derivative *racR*::Cm + pMA1-1261-1263	This study
*C. jejuni racRS+*p	81116 derivative *racRS*::Cm + pMA1-1261-1263	This study
*C. jejuni* 11168	Wildtype	Palmer et al. (1983)
*C. jejuni 81-176 ggt*	81-176 derivative *ggt*::Cm	This study
**Plasmids**
pBluescript II KS	Ap^R^; 3.0 kb; LacZα	Stratagene
pGEM-T Easy	Ap^R^; 3.0 kb; LacZα; TA cloning vector	Promega
pT7.7	Ap^R^; 2.5 kb; expression vector	Tabor and Richardson (1985)
pMA1	Km^R^; 10 kb; *E. coli/C. jejuni* shuttle vector	van Mourik et al. (2008)
**Plasmids constructed for mutagenesis**
pUWM799	Ap^R^; 4.2 kb; pBluescript II KS/internal fragment of *C. jejuni ggt*	This study
pUWM804	Ap^R^ Cm^R^; 4.9 kb; pBluescript II KS/*ggt::*Cm	This study
pGEM-1261-1263	Ap^R^; 5.5 kb; pGEM-T Easy/*C. jejuni 1261-1263*	This study
pGEM1261::Cm	Ap^R^ Cm^R^; 6.3 kb; pGEM-T Easy/*racR::*Cm	This study
pGEM1262::Cm	Ap^R^ Cm^R^; 6.3 kb; pGEM-T Easy/*racS::*Cm	This study
pGEM1261-62::Cm	Ap^R^ Cm^R^; 5.3 kb; pGEM-T Easy/*racRS::*Cm	This study
**Plasmids constructed for complementation**
pMA1-1261-1263	Km^R^;12.5 kb; pMA1/*Cj1261-1263*	van der Stel et al. (2015)

### Construction of *C. jejuni ggt, racS, and racRS* Mutants

To inactivate the *ggt* gene, the *ggt* gene was amplified by PCR using the oligonucleotides Cjj67Sac and Cjj67Xba (**Table 2**). The obtained PCR product was digested with *Cla*I and *Pst*I resulting in a 1.2-kb DNA fragment, which was ligated into pBluescript II KS to give plasmid pUWM799. Plasmid pUWM799 was digested with *Bgl*II to remove a 0.1-kb internal *ggt* fragment and was ligated to a 0.8-kb *Bam*HI fragment containing the *cat* cassette (0.8 kb) of pRY109. The resulting *ggt* knockout construct pUWM804 contained the *cat* cassette in the same orientation as the *ggt* gene.

**Table 2:** Oligonucleotides used in this study.

Name	Sequence (5′-3′)^a^
Cjj67Sac	CGCGAGCTCGCTTTTTGCGGTGGTAGG
Cjj67Xba	AGTTCTAGAGGAGATCCTGTGCCTGTG
GGT204	AAAGCATGCATTGCACTTTCAATAAATTTTAAATATTTTAGC
GGT104	AAAGCATGCATAAAATGAGAATATTTGATAC
GGT69	AAAGCATGCTTTAAATATTTTTATAAAAATATATC
GGT35	AAAGCATGCAATTTATCAATACCCCTAGTTTTG
GGT28	AAAGAGCTCCATTTTACTCCTTTTTAATGATATATAG
**Primers for gel mobility shift assay**
GGTprR	GCTTCAAATTTCATATTGCACTT
GGTprFDIG	TTGAAATCGCAAATATAGCT
Cj200RDIG	GTTTTAGACTATCTGCAAAA
Cj201F	TTTCATCTTCAATATACTCTAA
CJ0145RDIG	TTAAAAACAATCTTCTTTCCAT
CJ0145F	TTTCTAGTACAGTAAGTGATATAGC
**Primers used for real-time RT-PCR**
ggtftaq	TGCGAGTTATGGTTCAGGTG
ggtrtaq	TTAGCTTCTCCGCCTACAAG
gltBftaq2	ACACGATGCCTGTGGTATCG
gltBrtaq2	TCGGTGTTCAAGATTCATCAAAAT
aspAftaq	TATGGGATAAGCATAGTGAAGTTCAAG
aspArtaq	CGCTTTAATAATCGCATCTTGGA
rpoDftaq	GAACGAATTTGATTTAGCCAATGA
rpoDrtaq	TGTCCCATTTCTCTTAAATACATACGA

*^a^Restriction sites introduced for cloning purposes are underlined.*

To construct the *racS* and *racRS* knockout constructs plasmid pGEM1261 (van der Stel et al., 2015), containing the genes Cj1261–Cj1263 was amplified by PCR using the primers RacSendBamHI/RacSstart2BamHI or Cj1261FBamHI/RacSendBamHI, respectively. The resulting PCR products were digested with *Bam*HI and ligated to a 0.8-kb *Bam*HI *cat* cassette from pAV35 (Vliet et al., 1998), resulting in plasmids pGEM1262::Cm and pGEM1261-1262::Cm, respectively.

The genes *ggt, racS*, and the *racRS* were disrupted in *C. jejuni* strain 81116 or 81-176 by natural transformation using the knock-out plasmids pUWM804, pJET1262::Cm and pGEM1261-1262::Cm, respectively. Double cross-over recombination events were confirmed by PCR.

### Construction of *racRS* Complementation Strain

To complement the *racRS* mutant, the complementation plasmid pMA1–1261–1263 (van der Stel et al., 2015) was first transformed into *E. coli* S17 and then conjugated (Labigne-Roussel et al., 1987) to the *racRS*::Cm mutant strain.

### Purification of Recombinant RacR and Cytoplasmic RacS

RacR (N-His) and RacScyto (N-His) were expressed and purified as described before (Wösten et al., 2004) using plasmids pT7.7-RacR(N-his) and pT7.7-RacScyto(N-his). Protein concentrations were determined using the BCA protein assay kit (Pierce).

### Construction of *ggt* Promoter Luciferase Constructs

To localize where RacR binds on the *ggt* promoter, different lengths of the upstream region of the *ggt* gene were amplified by PCR. The PCRs were generated by fusion polymerase (Thermo) with *Cj*81116 genomic DNA as template and one of the primer pairs GGT28/GGT204, GGT28/GGT104, GGT28/GGT69, or GGT28/GGT35. The PCR products were digested with *Sac*I and *Sph*I (Thermo) and cloned into *Sac*I, *Sph*I digested plasmid pMA5-metK-luc (Bouwman et al., 2013) to replace the *metK* promoter. To obtain a promotorless luciferase vector, pMA5-metK-luc was digested with *Sac*I and *Sph*I, blunted with the blunting enzyme from the CloneJET PCR Cloning Kit (Thermo) and finally self-ligated. The plasmids were verified by sequencing (Macrogen). The obtained plasmids were transformed to *E. coli* S17 and subsequently conjugated to *C. jejuni* 81116 and the *racRS* mutant.

### Real-Time RT-PCR

Total RNA was extracted from late logarithmic phase *C. jejuni* cultures grown in HI medium at 42°C under oxygen limiting conditions with the addition of 50 mM nitrate, using RNA-Bee^TM^ kit (Tel-Test, Inc) according to the manufacturer’s specifications. Real-time RT-PCR analysis was performed as previously described (van der Stel et al., 2015). Primers used in this assay are listed in **Table 2**. Experiments were repeated with three independently grown cultures. Fold increase was calculated with the 2^ΔΔCt^ method (Schmittgen and Livak, 2008) using *rpoD* as reference gene.

### GGT Activity Assay

To assay the GGT activity the production of 3-carboxy-4-nitroaniline was followed by measuring the absorbance at 405 nm according to a modified procedure described by (Chevalier et al., 1999). Briefly, 1 mL of bacterial culture was pelleted and stored at −80°C for at least 1 h. The pellet was resuspended in 250 μL buffer A (50 mM Tris/HCl (pH 7.6), 1 μg/mL lysozyme), and incubated for 30 min on ice. Next, the bacteria were disrupted by sonication followed by centrifugation (10 min at 12000 × *g* at 4°C). From the cell free bacterial lysate 20 μl was mixed together with 180 μl of a reagent containing 2.9 mM L-γ-glutamyl-3-carboxy-4-nitroanilide, 100 mM glycylglycine and 100 mM Tris-HCl (pH 8.2). Samples were measured every 60 s during an incubation period of 30 min at 37°C. From these graphs the slope of all values in a linear range was calculated. Protein concentration was determined using the BCA method (Pierce). GGT activity is expressed as nmol min^-1^ mg protein^-1^. The data shown represents at least three independent experiments.

### Luciferase Assay

Expression of the luciferase in *C. jejuni* 81116 and *racRS* mutant strain harboring the pMA5-ggtprom-luc plasmids was measured as previously described (Bouwman et al., 2013). Briefly, overnight cultures were diluted to an OD_550__nm_ of 0.05 and grown for 7.5 h in HI with 50 mM KNO_3_ in an oxygen limiting atmosphere at 37°C. One milliliter of each culture was pelleted (8000 ×*g*, 5 min, 4°C) and suspended in 100 μL RLB buffer (Promega) supplemented with 0.5% Triton-X100. Suspensions were stored at −80°C for at least 30 min to disrupt the bacteria. Bacterial lysate (20 μL) was mixed with 50 μL of luciferase reagent (Promega) and RLU’s were measured immediately on a luminometer (TD20/20, Turner Designs). The data shown represents at least three independent experiments.

### Gel Mobility Shift Assay

The promoter regions upstream of *ggt*, *phoX* (Cj0145), and Cj0200c were amplified by PCR using Dreamtaq polymerase (Thermo) and one of the primers sets GGTpromDIG/GGT204, GGTpromDIG/GGT104, GGTpromDIG/GGT69, GGTpromDIG/GGT35, Cj145F/CJ0145RDIG, and Cj200F/Cj200RDIG, respectively, (**Table 2**) and *Cj*81116 chromosomal DNA as template. Primers GGTpromDIG, Cj0145RDIG, and Cj200RDIG were ordered with a digoxigenin (DIG) label (Eurofins genomics). Approximately, 50 fmol DIG-labeled PCR fragments was incubated with His-tagged RacR, RacScyto, and 2 mM ATP for 30 min on ice. RacR, RacScyto, and ATP were preincubated for 5 min at 37°C to allow phosphorylation. The binding buffer used for protein-DNA incubations was 20 mM Tris-HCl (pH 7.4), 5 mM MgCl_2_, 100 mM KCl, 100 μg/ml bovine serum albumin, 1 μg/ml poly-(dI-dC) and 10% glycerol. Samples (10 μl) were run on a 4% non-denaturing Tris glycine polyacrylamide gel at 4°C. After electrophoresis the DNA was blotted on a hybond-N+ membrane (Amersham) and PCR fragments were visualized using α-DIG-AP, Fab fragments, and CSPD substrate (Roche).

### Growth Curves

*Campylobacter jejuni* precultures were grown for 7 h in HI medium with the addition of 50 mM nitrate at oxygen limiting conditions at 42°C. The precultures were diluted 50 times in 300 μL DMEM (without glucose, glutamine, pyruvate, bicarbonate, and phenol red, D5030, Sigma) with the addition of 10 mM TMAO and 10 mM glutamate, glutamine or aspartate, and growth curves were generated at 42°C in a 100 well honeycomb plate which was continuously shaking in a Bioscreen C MRB (Oy Growth Curves Ab) computer-controlled incubator. The incubator was placed inside an anaerobic chamber (Coy Labs, Grass Lake, MI, USA), due to suboptimal gas exchange in the honeycomb plate the oxygen concentration was set to 1%, which yielded comparable growth as when bacteria were grown in rectangular flasks inside an anaerobic jar containing 0.3% O_2_. The OD_600nm_ of cultures was recorded every 15 min during 45 h. For clarity reasons only point at 2.5 h, or 5 h intervals are shown. Experiments were repeated three times in duplicate.

### Statistical Analysis

Prism software (GraphPad, San Diego, CA, USA) was used for statistical analysis. Results are shown as mean ± SEM. Data was analyzed by one-way ANOVA, followed with Bonferroni *post hoc* tests; *P* < 0.05 was considered statistically significant.

## Results

### RacR Regulates *ggt* Transcription and Activity

**FIGURE 1:**
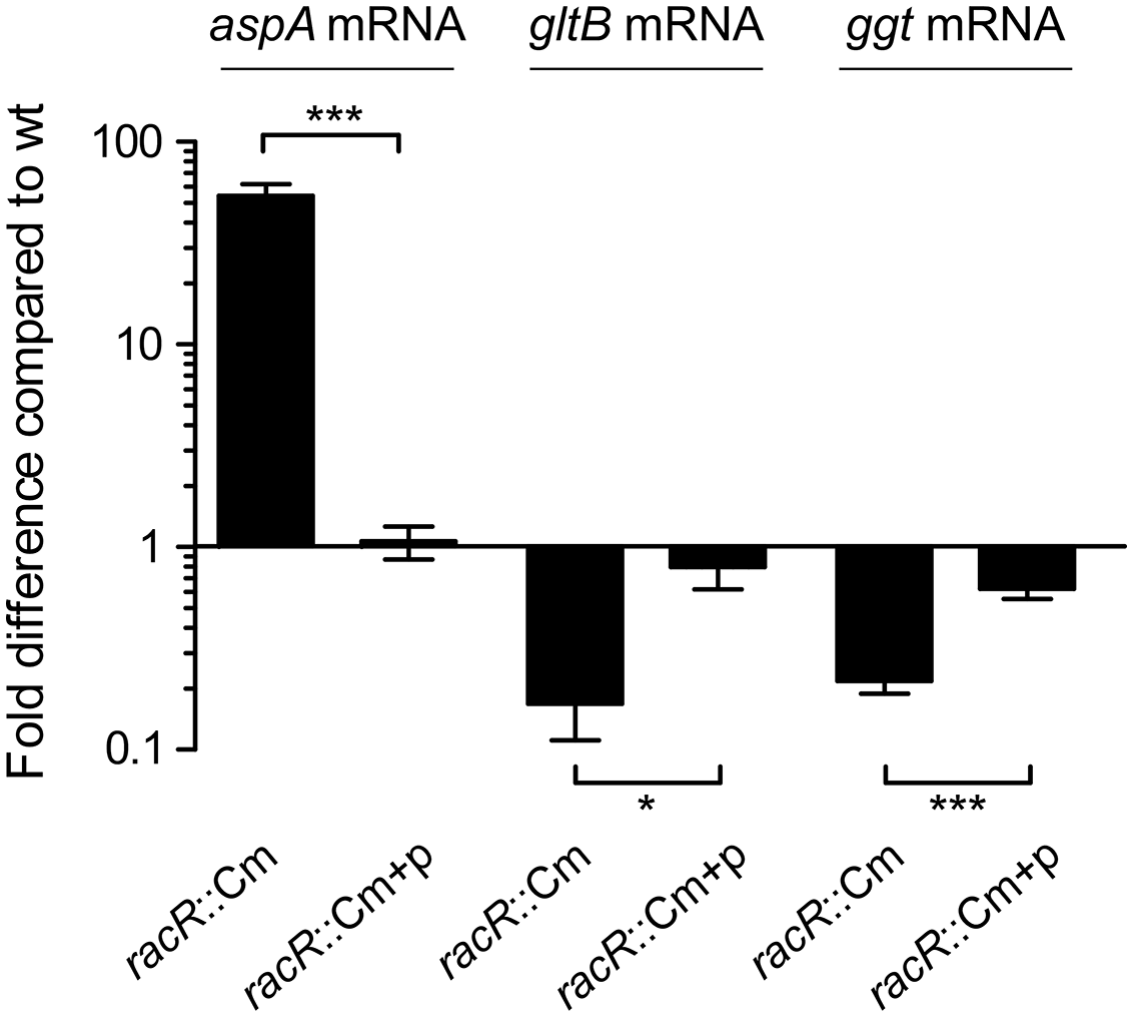
**RacR activates *ggt* transcription**. Real-time RT-PCR data showing the transcript fold difference of the *aspA, gltB*, and *ggt* mRNA in the *Campylobacter jejuni racR* mutant (*racR*::Cm) and complemented strain (*racR*::Cm+p) compared to the wt. Cultures were grown in Heart Infusion broth (HI) medium with addition of nitrate until end-log phase under oxygen limiting conditions. Fold increase is calculated using the 2^ΔΔCt^ method using *rpoD* as reference gene. Data represent the mean values and SE of three independent experiments. *p < 0.05, ***p < 0.001.

As the *C. jejuni* RacRS two-component system regulates the cytoplasmic glutamate production by activating the *gltBD* genes (van der Stel et al., 2015), we wondered whether GGT, responsible for the periplasmic glutamate production, is also regulated by the RacRS system. To investigate this we measured the *ggt* transcripts in the *C. jejuni* 81116 wildtype strains, the isogenic *racR* mutant, and in the complemented *racR* mutant, grown until late log phase under RacRS inducing conditions, i.e., 0.3% O_2_ with 50 mM nitrate. To verify that we used RNA that was isolated under RacR inducing conditions, we also measured the transcripts of *aspA* and *gltB* genes. Using real time RT-PCR we observed that inactivation of *racR* resulted in a 55-fold increase of *aspA* mRNA, and a sixfold decrease of the *gltB* consistent with our previous results (van der Stel et al., 2015) and confirming that RacR is induced under these conditions. A significant fivefold decrease was observed for the *ggt* mRNA transcripts. The differences between the wt and the *racR* mutant were almost restored to wt levels by introducing complementation plasmid harboring the RacRS operon (**Figure 1**). These results suggest that RacR has a strong influence on the production of glutamate as it not only activates the transcription of the genes required for the cytoplasmic glutamate production (*gltB)*, but also the periplasmic production of glutamate (*ggt)*.

**FIGURE 2:**
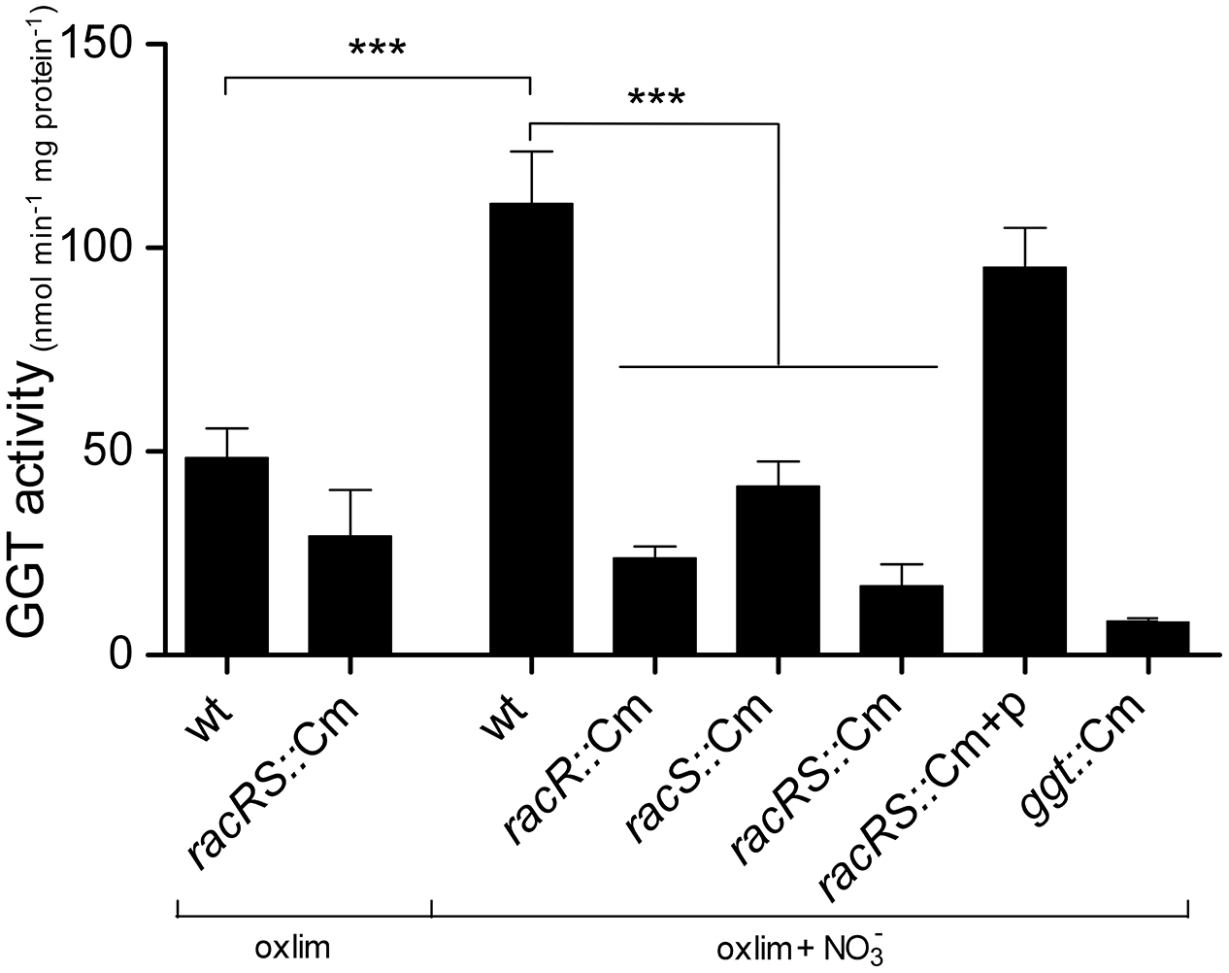
**RacR influences the γ-glutamyltranspeptidase (GGT) activity**. GGT activity was measured in cell lysate from stationary phase (20 h) cultures of wildtype 81116, the *racR, racS*, and *racRS* mutant strains, as well as the complemented *racRS* mutant, grown in HI medium at oxygen limiting conditions with or without the addition of nitrate. Mean and SE of at least three independent experiments are shown. ***p < 0.001.

To verify that the two-component system RacR/RacS also influences the GGT enzyme activity, we measured the GGT activity in *C. jejuni* stationary phase cultures grown at 0.3% O_2_ with or without the addition of nitrate (**Figure 2**). Only background levels of GGT activity were observed in the *ggt* mutant indicating that this enzyme is solely responsible for the production of 3-carboxy-4-nitroaniline in the GGT assay. Maximum GGT activity of bacteria grown in HI liquid medium was observed in stationary phase (data not shown). A low GGT activity was measured in wt and *racR* mutant when the strains were grown at 0.3% O_2_; however, the GGT activity increased threefold in the wt bacteria when nitrate was present. This induction was not observed in the *racRS* double mutant or the single *racR*, or *racS* mutant strains. When the *racRS, racR, or racS* mutants were complemented with a plasmid harboring the RacRS operon the GGT activity was restored to wildtype levels. These results indicate that GGT activity largely depends on a functional and activated RacR and RacS, as exists under limited oxygen condition in the presence of an alternative electron acceptor.

### RacR Protein Binds to the Promoter Region of the *ggt* Gene

**FIGURE 3:**
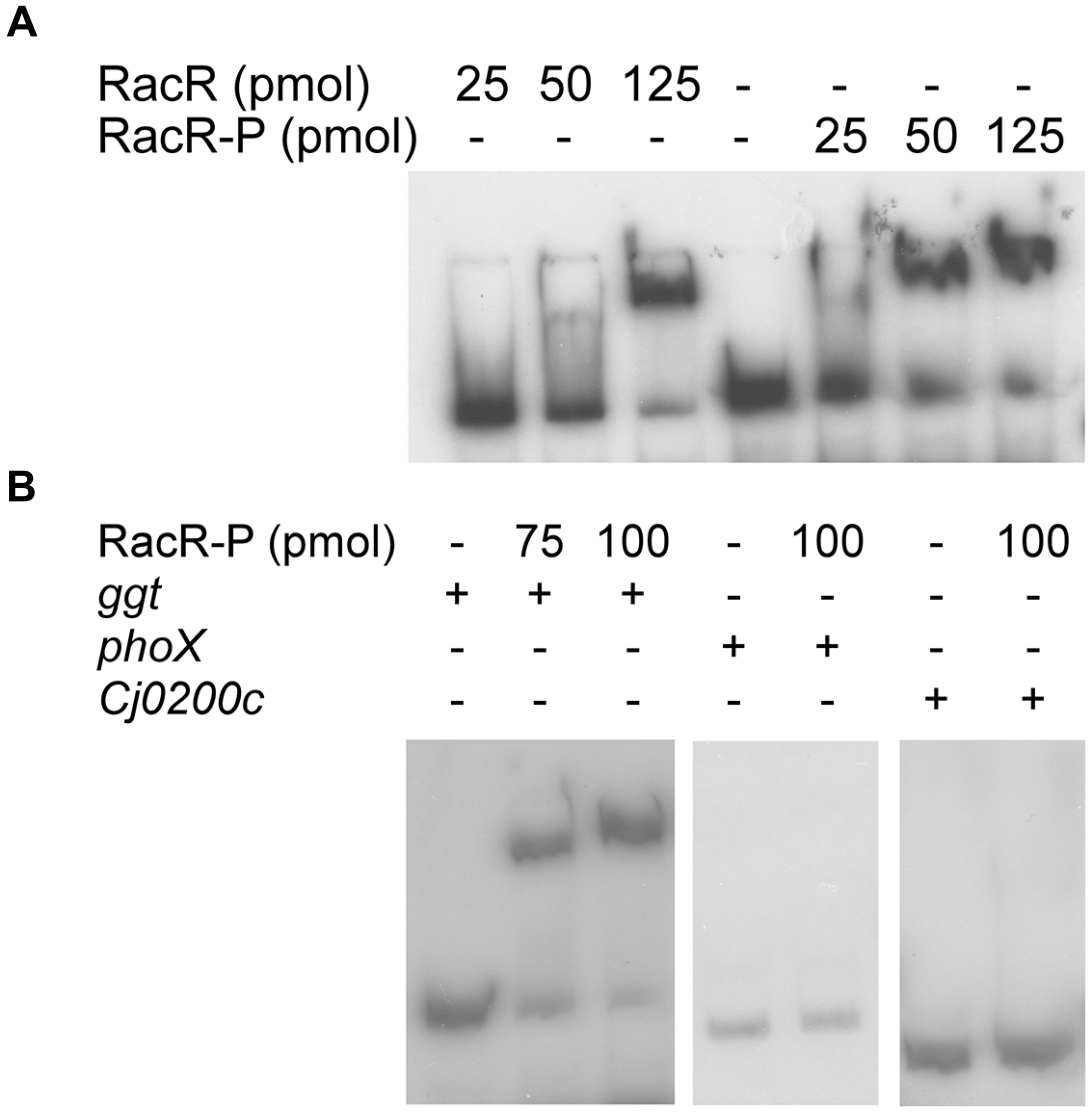
**RacR binds to the *ggt* promoter region as shown by electrophoretic mobility shift assays (EMSA)**. DIG-labeled PCR fragments (~50 fmol) containing the *ggt, phoX*, or Cj0200c promoter regions were incubated with RacR as indicated. **(A)** Influence of the phosphorylation of RacR on the binding to the *ggt* promoter. RacR was phosphorylated by RacScyto in the presence of ATP. **(B)** EMSA of the *ggt, phoX*, and Cj0200c promoter regions with phosphorylated RacR protein. The *phoX* and Cj0200c promoter regions were used as negative controls. RacR-P, phosphorylated RacR.

To investigate whether RacR activates the *ggt* gene directly by binding to the *ggt* promoter region, electrophoretic mobility shift assays (EMSA) were performed. Hereto the RacR response regulator and the cytoplasmic region of RacS were isolated as His-tagged recombinant proteins and together with ATP incubated with DIG-labeled DNA fragments containing the promoter region of the *ggt* gene or, as a negative control, the promoter regions of the *phoX* and *Cj0200c* genes. Incubation of RacR and the cytosolic region of RacS in the presence of ATP led to rapid phosphorylation of RacR (van der Stel et al., 2015). Unphosphorylated RacR bound to the *ggt* promoter but less RacR was needed when it was phosphorylated by the cytoplasmic part of RacS (**Figure 3A**). Phosphorylated RacR did not bind to the *phoX* and *Cj0200c* promoter fragments as no band shifts were observed, while a clear band shift was seen for the *ggt* promoter fragment (**Figure 3B**). These results indicate that RacR specifically binds to the *ggt* promoter and that phosphorylation of RacR enhances the binding affinity.

### RacR Binds to a RacR Binding Consensus Sequence in Front of the *ggt* Promoter

**FIGURE 4:**
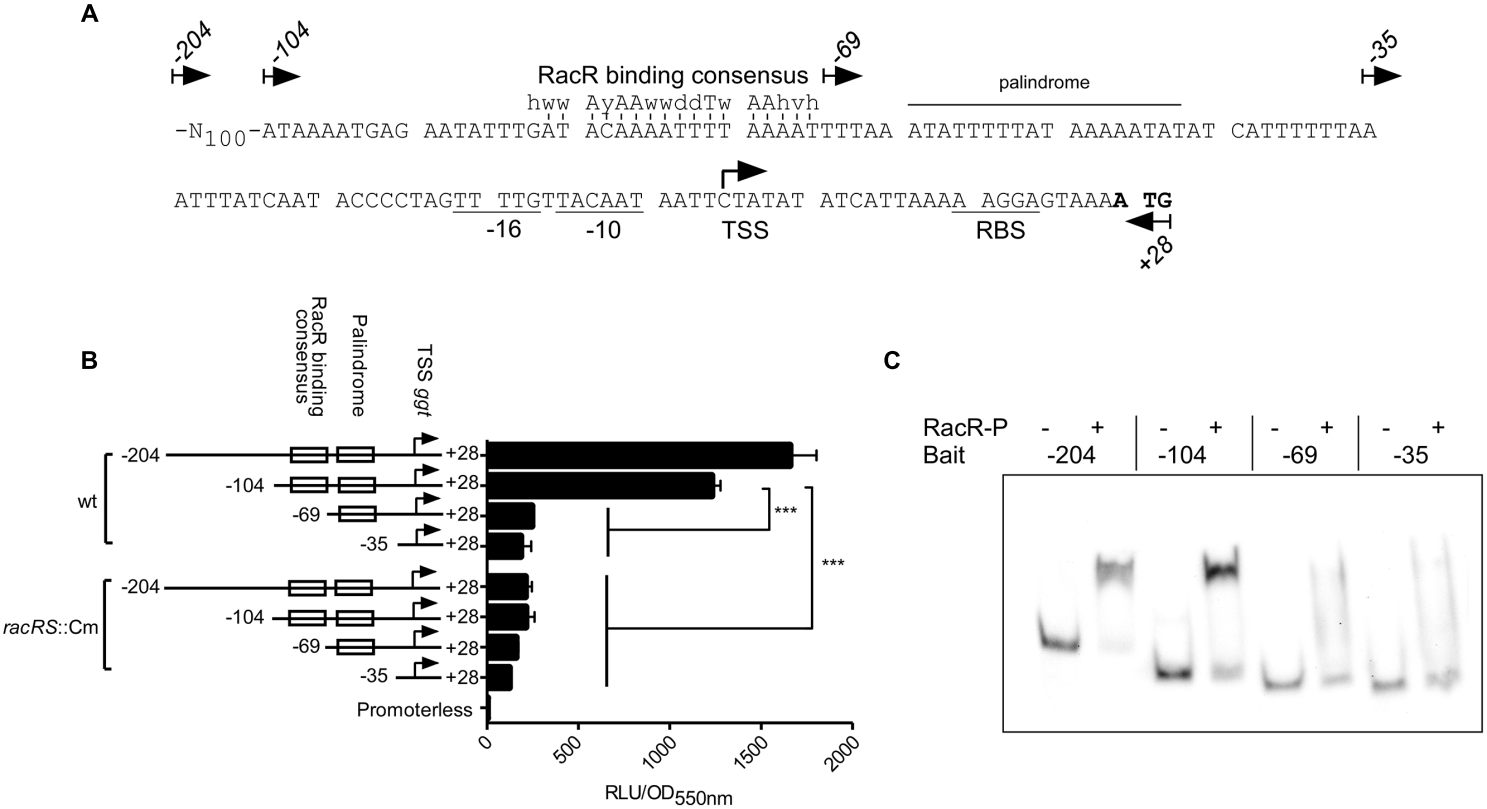
**RacR binds to a specific region on the *ggt* promoter. (A)** Nucleotide sequence and features of the *ggt* promoter. The start codon ATG is indicated in bold face, the putative -10 and -16 regions and ribosomal binding site (RBS) are underlined. A palindromic sequence is indicated with a horizontal bar. The previously identified RacR binding consensus sequence (van der Stel et al., 2015) is indicated above the predicted RacR binding site, vertical lines indicate matching nucleotides. Arrows indicate the 5′ termini and direction of the primers used to generate the *ggt* promoter elements for the luciferase reporter plasmids and EMSA bait DNA. The transcriptional start site of the *ggt* gene identified by (Dugar et al., 2013) is indicated with a hooked arrow. **(B)** Luciferase activities using different lengths of the region upstream of the *ggt* gene are determined in wt and *racRS*::Cm mutant bacteria. Cultures were grown until late-log phase at oxygen limiting conditions with the addition of nitrate. Data represents the mean and SE of three independent experiments. **(C)** EMSA experiments using the different *ggt* promoter elements. DIG-labeled PCR fragments (~50 fmol) were mixed with or without 50 pmol RacR and 25 pmol RacScyto in the presence of ATP. RacR-P phosphorylated RacR.

To investigate where RacR binds on the *ggt* promoter, an *in silico* analysis of the promoter region was performed (**Figure 4A**). Based on this analysis, primers were designed to amplify DNA fragments in order to study the different elements on the *ggt* promoter region. Besides the full intergenic region between the *ggt* and *c8j_0034* gene (-204 nt fragment) three truncated *ggt* promoter elements were generated; (1) a −104 nt fragment containing a putative RacR binding consensus sequence, a palindromic sequence and the putative −35, −16 and −10 region; (2) a −69 nt fragment lacking the putative RacR binding consensus sequence, and (3) a -35 nt fragment only containing the putative −35, −16 and −10 regions. These promoter elements were cloned in front of the luciferase reporter gene, replacing the *metK* promoter located on plasmid pMA5-metK-luc (Bouwman et al., 2013). Luciferase activity was measured in the wt and *racRS* mutant strain under RacR inducing conditions. Because of poor stability of the luciferase enzyme at 42°C (data not shown), all luciferase reporter assay experiments were performed at 37°C. In wt bacteria high luciferase activity was measured only from the promoter elements −204 and −104, both containing the predicted RacR nucleotide binding site (**Figure 4B**). All promoter fragments resulted in a low luciferase activity in the *racRS* mutant, which was, however, still higher than the luciferase activity of the strain carrying a promoterless luciferase plasmid. These results indicate that the region upstream of the *ggt* gene containing the predicted RacR consensus sequence is important for enhancing of the *ggt* transcription in a RacRS dependent manner.

In order to verify the luciferase reporter results, the different *ggt* promoter elements were subjected to EMSA experiments (**Figure 4C**). The -204 and -104 *ggt* promoter elements, harboring the predicted RacR binding site showed a distinct bandshift when phosphorylated RacR was present. In accordance with the luciferase assay results RacR did not bind to the two shorter fragments. These results prove that phosphorylated RacR binds to the upstream region of the *ggt* promoter region containing the predicted RacR binding consensus nucleotide site.

### RacR is Important for *C. jejuni* to Generate More Biomass out of Glutamine, under RacRS Inducing Conditions

**FIGURE 5:**
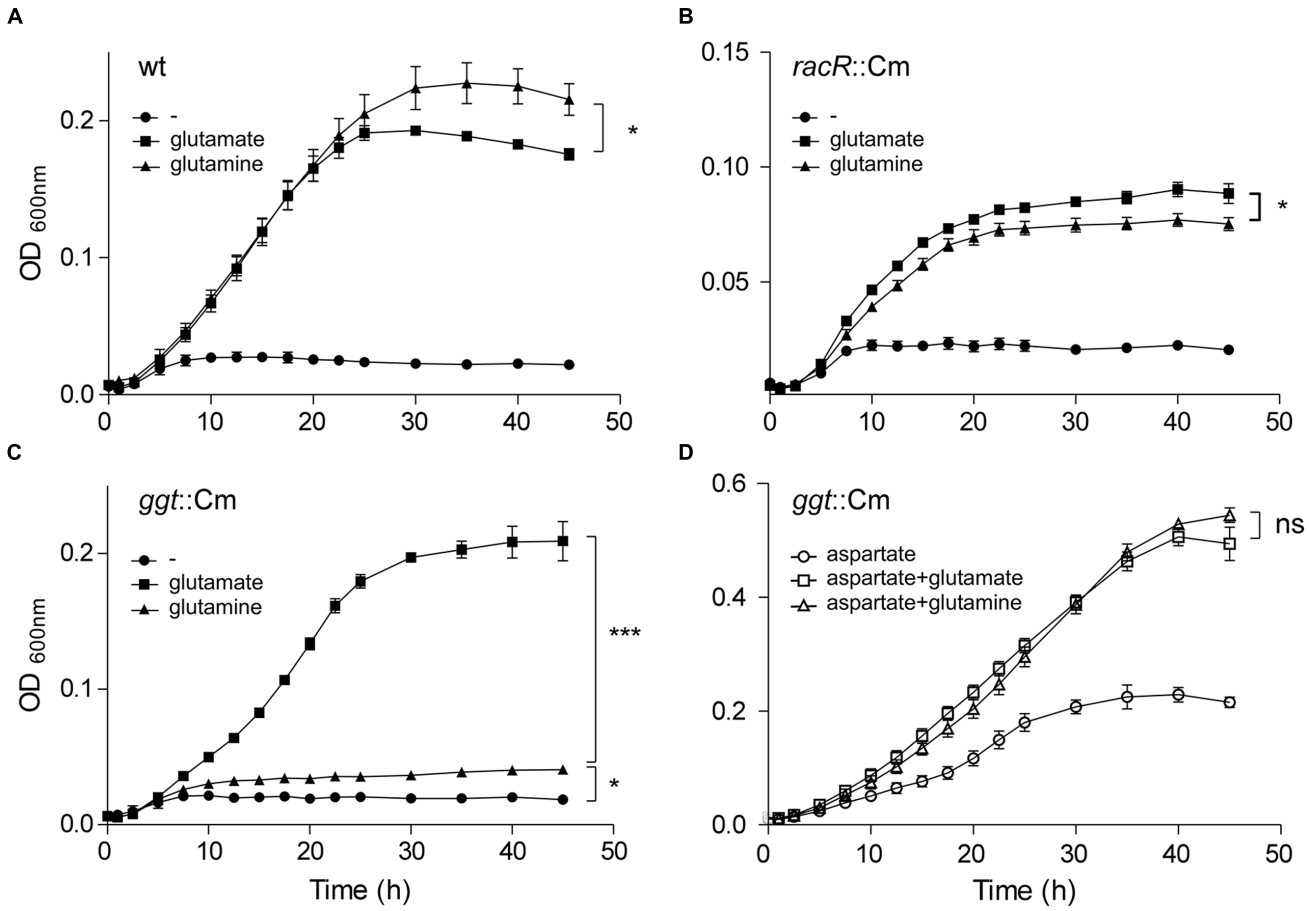
**The RacRS system is important for the generation of glutamate out of glutamine**. Growth curves were generated of the wt 81116 **(A)**, the *racR* **(B)**, and *ggt* **(C)** mutants grown in DMEM (circles) or with the addition of 10 mM glutamate (squares) or 10 mM glutamine (triangles) at 1% O_2_ with the addition of 10 mM TMAO as electron acceptor. **(D)** The same as **(C)** but with the extra addition of 10 mM aspartate to the medium. Data represent the mean and standard error of three independent experiments. Significance was calculated using the maximally obtained OD_600nm_ values. ns, not significant; *p < 0.05, ***p < 0.001.

To investigate whether the GGT activity contributes to an increased bacterial fitness, growth curves in DMEM medium with or without 10 mM glutamate or 10 mM glutamine were recorded for the wt, the *racR* and *ggt* mutant strains under RacR inducing conditions. To facilitate growth TMAO was used as electron acceptor, because nitrate proved to be detrimental for growth at these nutrient poor conditions. The growth rates of the wt grown with either glutamate or glutamine as sole carbon source were very similar, although the maximum OD_600__nm_ was higher when glutamine was present (**Figure 5A**). The *racR* mutant strain on the other hand showed a reduced growth rate when grown on glutamine compared to glutamate and consistently reached a slightly lower OD_600__nm_ (**Figure 5B**). Furthermore compared to the wt, the *racR* mutant shows a reduced growth rate and a lower maximum growth yield. The *ggt* mutant strain grew comparable to the wt on glutamate, but hardly grew on glutamine (**Figure 5C**), verifying that GGT is needed to utilize glutamine as sole carbon/energy source (Hofreuter et al., 2006). These results prove that under low oxygen conditions in the presence of alternative electron acceptors the RacRS system is important for the conversion of glutamine to glutamate.

### *Campylobacter jejuni* is Capable of Utilizing Glutamine in a GGT Independent Manner

Although GGT is required for growth with glutamine as sole carbon source, we observed that the *ggt* mutant reached a higher OD_600nm_ when grown on DMEM plus glutamine compared to DMEM alone (**Figure 5C**). *C. jejuni* possess a glutamine transporter PaqPQ that has been shown to be functional under nutrient rich conditions (Lin et al., 2009). To investigate whether the *ggt* mutant is able to grow on glutamine under nutrient rich conditions, aspartate was added to the DMEM medium. Both the growth rate and bacterial yield of the *ggt* mutant increased with glutamate and glutamine compared to aspartate alone (**Figure 5D**). Similar results were obtained when serine was used instead of aspartate (data not shown). This GGT independent glutamine utilization phenotype was also seen for the *C. jejuni* 81-176 *ggt* mutant strain and the *C. jejuni* 11168 strain, which naturally lacks the *ggt* gene (data not shown). These results indicate that GGT is important when other energy sources than glutamines are not available.

## Discussion

The highly conserved enzyme GGT in prokaryotes plays a key role in the glutamine and glutathione metabolism. However, the regulation of the transcription of this gene is poorly understood. Here we identified the response regulator RacR as the first prokaryotic transcription factor that directly regulates *ggt* gene transcription. The *C. jejuni* RacR activates *ggt* gene transcription under low oxygen conditions in the presence of alternative electron acceptors. The RacRS system therefore not only activates the cytoplasmic production of glutamate by upregulating the GOGAT system (van der Stel et al., 2015), but also influences the periplasmic production of glutamate by upregulating the GGT enzyme, and thus ensuring the use of extracellular glutamine as energy source under RacR inducing conditions.

Previously, the *ggt* gene was not identified as part of the RacR regulon as the micro-arrays used in that study were based on a *C. jejuni* strain that lacks the *ggt* gene (van der Stel et al., 2015). Only 31% of *C. jejuni* strains contain a *ggt* gene (de Haan et al., 2012). Because both RacR and GGT have been shown to be important for host colonization (Brás et al., 1999; Barnes et al., 2007; Hofreuter et al., 2008), we assumed that the RacRS system might also regulate the *ggt* gene. Real-time RT-PCR and GGT enzyme activity assays clearly showed that RacR activates the transcription of the *ggt* gene and GGT enzyme activity under limited oxygen conditions in the presence of alternative electron acceptors (**Figures 1 and 2**), the same result was observed in a *C. jejuni* 81-176 *racR* mutant (data not shown). Similar results were obtained in the *racRS* and the *racS* mutants, showing that both RacR and RacS are needed to activate *ggt* transcription.

Unequivocal evidence that *ggt* is a member of the RacRS regulon was obtained from EMSA and luciferase reporter assays (**Figures 3** and **4**). EMSA results showed that phosphorylated RacR strongly interacts with the *ggt* promoter, indicating direct regulation of *ggt* by RacR upon phosphorylation. The *C. jejuni* 81116 *ggt* promoter contains a conserved −10 and −16 regions but no −35 region (Petersen et al., 2003; Dugar et al., 2013). Apparently a strongly conserved −10 and −16 regions are not sufficient to activate the *ggt* transcription without additional transcription factors, as seen by real-time RT-PCR and luciferase assay (**Figures 1** and **4**). These results suggest that although a conserved consensus sequence for the −35 region of sigma 70 regulated promoters in *C. jejuni* is lacking the region upstream of the −16 region is essential to activate sigma 70 regulated promoters as has been observed already (Dugar et al., 2013; Salamasznska-Guz et al., 2013). Using the previously obtained RacR binding consensus (van der Stel et al., 2015), a potential RacR binding site was found ~80-bp upstream of the transcriptional start site (TSS) (Dugar et al., 2013), besides that, a palindromic sequence was found ~60-bp upstream of the TSS, which could be a potential regulatory element. Different lengths of the *ggt* promoter cloned in front of a promoterless luciferase reporter gene and EMSA experiments showed that only the fragments containing the predicted RacR binding nucleotide sequence were activated in the luciferase assay and band shifted in the EMSA experiment. This result clearly shows that the *ggt* gene belongs to the RacRS regulon.

Although present in many bacterial species, knowledge regarding the regulation of the GGT is limited. In *E. coli* and *B. subtilis* GGT activity is maximal in stationary growth phase (Tabor and Richardson, 1985; Xu and Strauch, 1996), while in *H. pylori*, an organism closely related to *C. jejuni*, *ggt* transcription is growth phase independent (Wachino et al., 2010). In *C. jejuni* the highest GGT activity measured at microaerophilic conditions is seen on plates in logarithmic growth phase (Barnes et al., 2007), however, when we used HI liquid medium the highest GGT activity was observed in stationary growth phase, independent of the oxygen concentration (data not shown). Nutrient availability might explain this difference as nitrogen-limiting conditions has been shown to activate the *B. subtilis* GGT (Kimura et al., 2004). So far, only in *B. subtilis* a transcription factor ComA has been identified to indirectly regulate the *ggt* gene. ComA of the quorum sensing two-component system ComP/ComA activates the *ggt* transcription, but has no influence on the *gltBD* genes (Ogura et al., 2001). The *C. jejuni* RacRS system is therefore unique that it directly regulates the periplasmic as well as cytoplasmic glutamate production in response to the available electron acceptors.

The GOGAT activity in other bacteria is strictly regulated, based on carbon, nitrogen, and energy status of the cell (Ninfa and Jiang, 2005), also because it requires the high energetic cofactor NADPH to generate glutamate. GGT does, however, not require high energetic co-factors; this could be a reason why it is expressed by *C. jejuni* in late log/stationary phase to scavenge for nutrients when energy levels are low.

**FIGURE 6:**
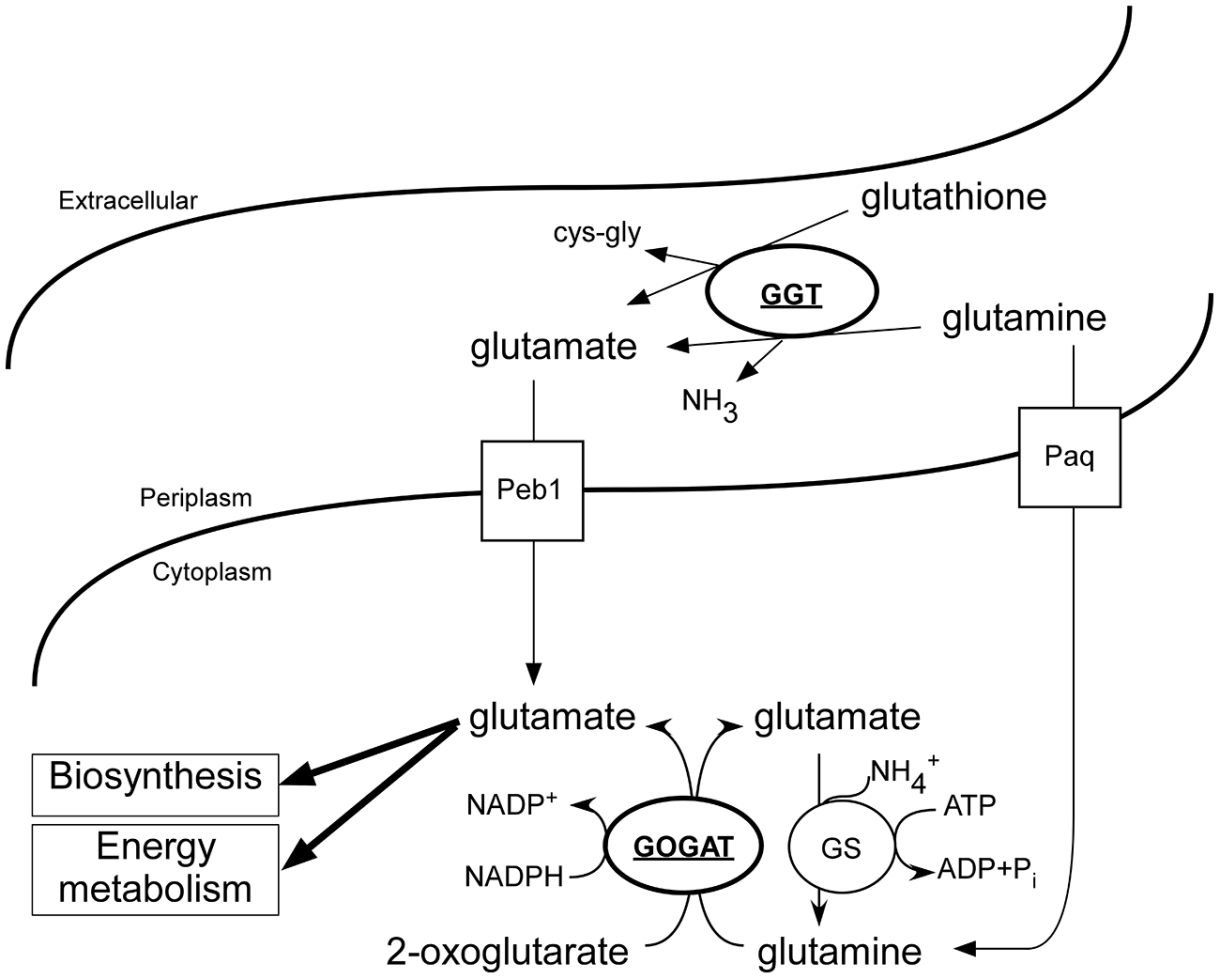
**Overview of the *C. jejuni* glutamate generating enzymes and involved transport systems**. Under low oxygen conditions in the presence of additional electron acceptors, RacR is activated and enhances the production of GGT and GOGAT (bold and underlined). Glutamine and glutathione are converted to glutamate in the periplasm by GGT; glutamate is consequently imported and used as carbon/energy source and anabolic precursor. Independent of GGT, glutamine can be imported and converted to glutamate by GOGAT, however, this process is dependent on the presence of additional carbon/energy sources.

The role of GGT varies among organisms, in animals the enzyme is used to recycle glutathione, while in yeast cells GGT is used as metabolic enzyme to utilize nitrogen sources (Mehdi and Penninckx, 1997). In *H. pylori* it was shown that an ammonia generating cycle consisting of periplasmic GGT and AnsA is present that aids in acidic resistance (Miller and Maier, 2014) and is essential for colonization and pathogenicity. The role of GGT in *C. jejuni* has been proposed to be metabolic, necessary for the acquisition of energy, carbon, and nitrogen, by deamination of glutamine, or acquisition of additional sulfur by metabolizing glutathione (Hofreuter, 2014; Vorwerk et al., 2014). Furthermore, GGT has been shown to be important for the pathogenesis of *C. jejuni*, by inhibiting cell proliferation and causing apoptosis (Barnes et al., 2007; Floch et al., 2014). Here we show that *ggt* is co-regulated with other metabolic genes in *C. jejuni* by RacR (**Figure 1**). In the periplasm GGT converts glutamine and glutathione to glutamate (**Figure 6**), which is subsequently transported to the cytoplasm via glutamate transporter PEB1 (Del Rocio Leon-Kempis et al., 2006). Up-regulation of *ggt* yields a growth advantage when grown on glutamine, confirming a metabolic role for GGT. This growth advantage is, however, only observed when other more favorable nutrients are less available (**Figure 5D**). When other carbon sources are present, the transporter PaqPQ (Lin et al., 2009) probably imports glutamine, which is converted to glutamate by the GOGAT system. Recently it has been reported that most Campylobacter cow isolates lack the *ggt* gene, while *ggt* is common in poultry and human isolates (de Haan et al., 2012). *C. jejuni* clades that lack *ggt* often harbor the fucose utilization gene cluster, while co-occurrence is rarely observed (de Haan et al., 2012), which could explain why strains lacking *ggt* are able to colonize chickens while a constructed *ggt* mutant can not. As humans and poultry are both omnivores, they consume a diet richer in protein than cattle. This results in a higher glutamine concentration in the gut (Stella et al., 2006), which is in favor of GGT possessing *C. jejuni* strains.

Overall, we show that the *C. jejuni* RacRS two-component system directly regulates the *ggt* gene transcription under limited oxygen conditions when alternative electron acceptors are present. The RacRS system is the first identified system that directly regulates both the periplasmic glutamate production (GGT) as well as the cytoplasmic glutamate production (GOGAT) and plays an important role in the metabolism of *C. jejuni.*

## Conflict of Interest Statement

The authors declare that the research was conducted in the absence of any commercial or financial relationships that could be construed as a potential conflict of interest.
